# Contributions of Mass Spectrometry to the Identification of Low Molecular Weight Molecules Able to Reduce the Toxicity of Amyloid-β Peptide to Cell Cultures and Transgenic Mouse Models of Alzheimer’s Disease

**DOI:** 10.3390/molecules24061167

**Published:** 2019-03-24

**Authors:** Raluca Ştefănescu, Gabriela Dumitriṭa Stanciu, Andrei Luca, Ioana Cezara Caba, Bogdan Ionel Tamba, Cosmin Teodor Mihai

**Affiliations:** 1 Center for Advanced Research and Development in Experimental Medicine (CEMEX), “Grigore T. Popa” University of Medicine and Pharmacy, 16 Universității Street, 700115 Iaşi, Romania; raluca.stefanescu@umfiasi.ro (R.S.); ioana-cezara.grigoriu@umfiasi.ro (I.C.C.); bogdan.tamba@umfiasi.ro (B.I.T.); cosmin-teodor.mihai@umfiasi.ro (C.T.M.); 2 Faculty of Pharmacy, “Grigore T. Popa” University of Medicine and Pharmacy, 16 Universităṭii Street, 700115 Iaşi, Romania

**Keywords:** Alzheimer’s Disease, amyloid-β peptides, mass spectrometry, proteolytic enzymes, anti-aggregating compounds, in vitro models, cell viability, transgenic mouse models, cognitive dysfunction, spatial working memory

## Abstract

Alzheimer’s Disease affects approximately 33 million people worldwide and is characterized by progressive loss of memory at the cognitive level. The formation of toxic amyloid oligomers, extracellular amyloid plaques and amyloid angiopathy in brain by amyloid beta peptides are considered a part of the identified mechanism involved in disease pathogenesis. The optimal treatment approach leads toward finding a chemical compound able to form a noncovalent complex with the amyloid peptide thus blocking the process of amyloid aggregation. This direction gained an increasing interest lately, many studies demonstrating that mass spectrometry is a valuable method useful for the identification and characterization of such molecules able to interact with amyloid peptides. In the present review we aim to identify in the scientific literature low molecular weight chemical compounds for which there is mass spectrometric evidence of noncovalent complex formation with amyloid peptides and also there are toxicity reduction results which verify the effects of these compounds on amyloid beta toxicity towards cell cultures and transgenic mouse models developing Alzheimer’s Disease.

## 1. Introduction

The large and continually increasing number of people affected by Alzheimer’s Disease (AD) led to an increasing number of scientific studies that aim to clarify the molecular mechanisms of this disease and to identify a noninvasive early diagnosis and treatment [1].

The presence in the brain of extracellular deposits and intraneuronal neurofibrillary tangles composed of aggregated amyloid-beta peptide and abnormally phosphorylated Tau protein respectively, are considered the most important histological changes observed post-mortem in the brain of Alzheimer’s Disease patients. Amyloid beta peptide (Aβ) is a 40 or 42 amino acid long peptide having the sequence D^1^AEFRHDSGYEVHHQKLVFFAEDVGSNKGAIIGLMVGGVVIA^42^ and is cleaved by β- and γ-secretase from one of the 3 major isoforms of the amyloid precursor protein (APP) namely APP_695_, APP_751_ and APP_770_. The former isoform is abundantly expressed in neuronal cells and in lower amounts in non-neuronal cells while the latter two isoforms are ubiquitously expressed [2]. Amyloid precursor proteins are type I transmembrane proteins containing a long extracellular domain, a short intracellular domain and a single-pass transmembrane domain. The cleavage of amyloid precursor proteins by β-secretase which occurs in the extracellular environment and by γ-secretase in the transmembrane domain yields in the extracellular medium the peptides Aβ(1–40) and Aβ(1–42). The quantitation of these two peptides in human plasma indicated a ratio of 10:1 in the case of healthy individuals and 7:3 in the case of people affected by the Familial Alzheimer’s Disease (FAD) [3,4,5]. Both peptides self-aggregate in aqueous solutions forming oligomers and fibrils which are toxic to the neuronal cells. The nucleation time decreases with increasing concentrations of Aβ(1–42) while the fibril elongation rate is higher for the pure Aβ(1–40) aqueous solution. The identification of the molecular ions corresponding to the oligomers and the determination of the cross section by ion mobility mass spectrometry allowed the identification of the oligomerization mechanism for these two peptides: Aβ(1–40) forms dimers and tetramers while Aβ(1–42) forms dimers, tetramers, hexamers and dodecamers [6].

**Figure 1:**
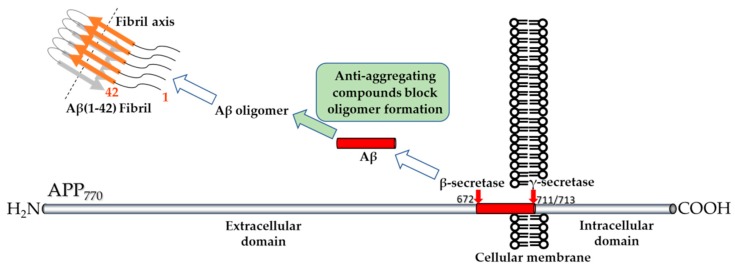
Schematic representation of the therapeutic strategy based on the finding of an Aβ aggregation inhibitor. Cleavage of the amyloid precursor protein (APP) containing 770 amino acids, by β-secretase in the extracellular domain after methionine-671 and by γ-secretase in the transmembrane domain after valine-711 and alanine-713 produces in the extracellular medium the free peptides Aβ(1–40) and Aβ(1–42). Both peptides form oligomers and fibrils. Anti-aggregating compounds which inhibit the oligomerization of the amyloid peptides are searched.

Current therapeutic strategies developed against AD include the identification of molecules able to form noncovalent complexes with the amyloid beta peptides and to increase their clearance from the organism affected by the disease (Figure 1) [7]. For understanding the mechanism underlying each noncovalent interaction and for establishing whether the ligand molecule blocks or enhances the oligomerization, mass spectrometry methods were developed. Among these methods, epitope mapping relies on the interaction between the immobilized antibodies that are resistant to proteolysis and the intact or enzymatically cleaved antigen followed by the mass spectrometric analysis of the dissociated antigen fragments upon addition of an acidic solution compatible with electrospray (ESI) or matrix assisted laser desorption and ionization (MALDI) ionization sources [8]. Additional results were obtained by analyzing the non-covalent complexes directly by nano-ESI/ion mobility mass spectrometry. 

For the in vitro research on AD, several cell lines were used: as primary culture cell lines derived from rodents, cells derived from cancer cells such as neuroblastoma and pheochromocytoma cells. More recently, the researchers have focused on utilizing induced pluripotent stem cells (iPSCs) to create an AD model in vitro by transfecting cells with genes associated with early-onset Familial AD, mutant presenilin 1 (PS1), presenilin 2 (PS2) or APP.

Human neuroblastoma (SH-SY5Y) has been used to generate an in vitro model for AD and other neurodegenerative disease by directing the SH-SY5Y cells into neuronal lineage using several differentiating factors. Fully differentiated SH-SY5Y cells have been previously demonstrated to express a variety of different markers of mature neurons including growth-associated protein (GAP-43), neuronal nuclei (NeuN), synaptophysin (SYN), synaptic vesicle protein II (SV2), neuron specific enolase (NSE) and microtubule associated protein (MAP), and to lack expression of glial markers such as glial fibrillary acidic protein (GFAP) [9]. 

Three important characteristics of *SH-SY5Y* cells should be considered when using these cells for in vitro studies [10]: (a) cultures include adherent and floating cells, both viable; (b) the parental differentiated *SK-N-SH* cells contained two morphologically distinct phenotypes: neuroblast-like cells and epithelial-like cells from which the cells with neuroblast-like morphology contain tyrosine hydroxylase (TH) and dopamine-β-hydroxylase that are a characteristic of catecholaminergic neurons, whereas the epithelial-like counterpart cells lack these enzymatic activities [11]; (c) *SH-SY5Y* cells can be differentiated to a more mature neuron-like phenotype that is characterized by neuronal markers. However, this model does not undergo all the processes which occur during the progression of AD in humans due to interaction between different cancer genes.

Immortal rat hippocampal cell lines are obtained from embryonic rat hippocampus, covering the need for cell lines derived from a known brain region origin that express phenotypes of particular subsets of cells. The cells are immortalized by retroviral mediated oncogene transduction using tsA58 and U19tsa alleles of simian virus 40 large tumour antigen. The lines morphologically differentiate to neuronal and/or glial phenotypes in culture, and express neural trophic factors known to be expressed in the hippocampus. These cell lines are thus useful for studies of development, plasticity and commitment in hippocampal lineages and for studies of receptor function and intracellular signalling pathways in mitotic versus postmitotic cells [12]. 

Human Induced pluripotent stem cells (iPSCs) used as models for AD have been generated from patients with APP mutations, including an E693 deletion mutation and APP gene duplication. Neurons carrying the E693 deletion case showed endoplasmic reticulum stress and oxidative stress. Also, intracellular Aβ oligomers were identified. Cells from patients with the APP duplication showed increased Aβ(1–40) peptide expressing increased phosphorylated-tau and increased active GSK3 beta. Models have also been generated using fibroblasts from patients with presenilin mutations, in PS1 and PS2. Cells with presenilin mutations had increased Aβ(1–42) secretion consistent with pathogenic hypotheses about AD. 

AD iPSCs have also been generated from patients with the V717I mutation in the APP gene. This mutation translated in dramatic increase of APP expression and Aβ levels as well as elevated β-secretase cleavage of APP, resulting in increased levels of both soluble APP-β and Aβ [13].

The use of *Mus musculus* as translational model organism to mimic human pathology is based on the strong genetic and physiological similitudes between rodents and humans. These models allow the testing of novel therapeutic strategies as well as the study of the disease progression and fundamental pathophysiology in a manner that is impractical or unethical in people.

In the study of neurodegenerative diseases translational (knock-in, knockout or transgenic) mice are valuable models because they have corresponding, although not identical, neuronal networks and neurobiological processes with humans. Furthermore, these in vivo models are inexpensive in matters of preserving and breeding or maintaining genetic control. Currently, early-onset Familial AD-associated mutations in amyloid precursor protein (APP), presenilin 1 (PS1) and presenilin 2 (PS2) were the most commonly used to facilitate the development of translational rodent models of AD. Nonetheless, it is necessary to consider that, none of these in vivo models of AD completely replicates the critical characteristics of the disease, represented by plaque and tangle formation, cognitive decline, synaptic loss, and neurodegeneration in a progressive manner [14].

Without a doubt, even with the above-mentioned limitations, translational AD models contribute to the enhancement of AD research by establishing the unique properties of each mutation and remain greatly useful for study of specific aspects associated with the disease appearance or progression. These models have become vital tools in improving the efficacy and safety profile of novel drugs and in surveying the temporal changes underlying the disease progression in a whole organism, studies that are typically far more limited in human subjects or tissues [15,16].

In this review, we aim to identify studies that assess the anti-aggregating properties and the ability to reduce Aβ toxicity of substances from different chemical classes both in vitro and in vivo. Our main focus was mass spectrometric analyses of the complexes between the substance and Aβ peptides as well as the identification of the Aβ segment which interacts with the substance. A comparison between the experimental conditions employed for the observation of the noncovalent complex in gas-phase and for the toxicity experiments carried out in vitro and in vivo was also performed.

## 2. Low-Molecular Weight Compounds Tested for Decreasing Amyloid-Beta Peptide Oligomerization and Toxicity 

### 2.1. Humanin Peptide (HN)

Humanin is a 24 amino acid peptide having the following sequence: MAPRGFSCLLLLTSEID LPVKRRA. It was identified for the first time by Hashimoto and coworkers in the occipital lobe of the brain of an AD patient. The neuroprotective effect of humanin is abrogated when the following amino acid replacements are introduced in the sequence: P3A, S7A, C8A, L9A, L12A, T13A, S14A, P19A. The replacement of serine 14 to glycine increases neuroprotection more than 1000-fold [17]. 

**Figure 2:**
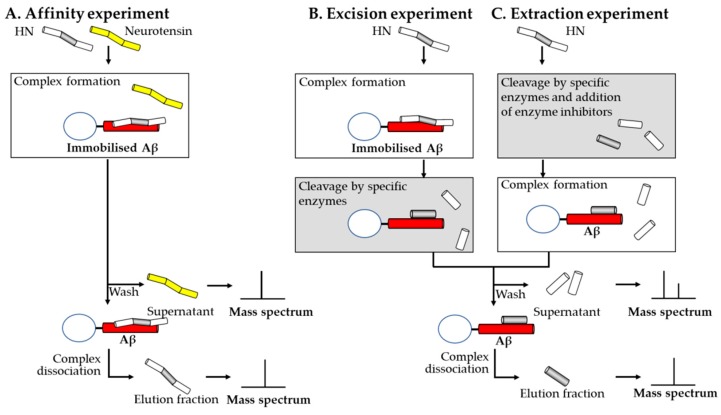
Schematic representation of the affinity mass spectrometric experiments carried out for the identification of the binding sequences of two peptides forming a complex using one immobilized binding partner. (**A**) Scheme of an affinity experiment using immobilized Aβ peptide. A mixture of humanin and neurotensin is incubated with the affinity medium. The mass spectrum of the supernatant should contain non-binding peptides and the mass spectrum of the elution fraction should contain the peptides presenting affinity towards amyloid-beta peptide. (**B**) In the excision experiment, intact humanin is incubated with immobilized Aβ followed by proteolytic cleavage of the peptide fragments that are not protected against proteolysis through the interaction. Mass spectrum of the supernatant reveals the regions from humanin which do not interact with Aβ while the mass spectrum of the elution fraction shows the region which is involved in the interaction. (**C**) In the extraction experiment humanin is first proteolitically cleaved, then protease inhibitors are added and the mixture is incubated with immobilized Aβ. The mass spectra of the supernatant and elution fractions obtained from both the excision and extraction experiments indicate the binding region of humanin.

Maftei and her collaborators established using mass spectrometry coupled to affinity chromatography methods that Aβ(1–40) and HN form a non-covalent complex and identified the sequence from both Aβ(1–40) and HN involved in the interaction [18]. They reported the semi-automated synthesis of the following peptides: humanin (HN), humanin (3–19), humanin preceded by an amino-terminal pentaglycine spacer (G5HN), humanin that possesses an amino-terminal pentaglycine spacer biotinylated at the amino-terminal end (BG5HN), humanin preceded by biotin and an amino-terminal pentaglycine spacer carrying one of the following mutations S14G, C8A, C8C(Acm) or C8S. Crude and purified peptides were analyzed using a MALDI-TOF Bruker Biflex II linear mass spectrometer in order to establish whether the correct peptide sequence was obtained. The formation of a non-covalent complex between HN and Aβ(1–40) was verified by immobilizing Aβ(1–40) on NHS-activated Sepharose and incubating the affinity chromatography medium obtained with an equimolar mixture of HN and neurotensin. The supernatant fraction collected after incubation and the elution fraction collected after washing the affinity medium and dissociating the complex with 0.1% trifluoroacetic acid were lyophilized, desalted using Zip-Tip and analyzed by MALDI-TOF. The presence of HN only in the elution fraction indicated that the HN bound specifically to Aβ(1–40) while neurotensin was present only in the supernatant, indicating that this peptide did not form a complex with Aβ(1–40). The authors analyzed using a nano-ESI-FTICR mass spectrometer, a solution containing C8A mutant peptide at 25 μM, Aβ (1–40) at 50 μM and 0.5 mM ammonium acetate (pH 6). In the mass spectrum, a molecular ion carrying five positive charges whose *m*/*z* value corresponds to the complex between C8A mutant peptide and Aβ(1–40) was observed. For the identification of the binding sequence of both humanin and Aβ(1–40) affinity columns were prepared, having Aβ(1–40) or humanin immobilized on NHS-activated Sepharose which allowed each affinity media to interact with the binding partner digested by trypsin, chymotrypsin or protease Glu-C prior to the addition in the affinity column or digested after the formation of the complex. The results indicated that the sequences HN(5–15) and Aβ(17–28) are involved in the interaction between humanin and Aβ(1–40) (Figure 2).

The neuroprotective effect of HN-S14G on the toxicity induced by Aβ(25–35) over PC12 (rat phaeochromocytoma) cells and its potential mechanisms were investigated. Incubation of PC12 cells with Aβ(25–35) (25 μM) caused cellular viability loss, decrease of mitochondrial membrane potential and increase in cytochrome C releases from mitochondria leading to cell apoptosis. All these toxic effects were inhibited by pretreatment with HN-S14G (100 nM) 6 h before exposure to Aβ(25–35): cell toxicity and apoptosis were reduced; mitochondrial membrane potential was stabilized and the release of cytochrome C from mitochondria was blocked. HN-S14G also ameliorated the Aβ(25–35)-induced Bcl-2/Bax ratio reduction (downregulation of Bcl-2 and upregulation of Bax) and decreased caspase-3 activity in PC12 cells. These findings indicate that mitochondria are involved in the protective effect of HN-S14G against Aβ(25–35) [19].

Both in vivo and in vitro studies sustained the protective effects of HN which seems to act as a signal peptide constraining neurotoxicity from several AD related insults. In vitro findings highlighted that HN might antagonize neurotoxicity induced by a wide range of FAD related proteins including APP, PS1, PS2, and Aβ peptides, such as Aβ(1–42) and Aβ(25–35) [20]. Similarly, in vivo evidence, risen from intracerebroventricular (icv) delivery of doses greater than 50 pmol of HN-S14G followed by Y-maze measurements, plea for proprieties against deterioration of short-term and spatial working memory caused by Aβ(25–35) (10 nmol/5 μL/site, icv) which was injected 21 days before testing [21].

Additionally, chronic administration of HN-S14G treatment (3-month, 0.1 μg/0.5 mL of HN-S14G diluted in normal saline solution, through ip delivery) significantly ameliorated spatial learning and memory deficits, reduced Aβ plaque accumulation and insoluble Aβ concentrations, decreasing neuroinflammatory responses in middle-age APPswe /PS1dE9 mice [22]. Niikura et al. [23] reported similar outcomes in APPswe, tauP310L, and PS1M146V triple transgenic mice (3×Tg-AD) following chronic intranasal instillation with 10 nmol of HN-S14G. With both total amount and phosphorylation status of tau unaffected, it appears that cytoprotective effect of HN is independent of tau pathology [23,24].

### 2.2. Tramiprosate

Martineau and his collaborators investigated the noncovalent interaction between tramiprosate and the following peptides: (i) Aβ(1–40), (ii) Aβ(1–42), (iii) Aβ(1–28) (iv) Aβ(1–28) all (−) carrying only negatively charged amino acids which contained the mutations D1G, E3S, R5G, D7A, K16E, D23S, K28S, and (v) Aβ(1–28) all (+) carrying only positively charged amino acids having the mutations D1G, E3S, D7A, E11S, E22S, D23A. 

Aβ peptide in 0.1% trifluoroacetic acid and deionized water together with tramiprosate in deionized water were mixed in order to achieve a final concentration of 30 μM and 150 μM. The pH of the resulting solution was adjusted to 7.4 using 0.1% NaOH. Mass spectrometric analyses of the complexes were carried out in positive mode using a ZQ 4000 mass spectrometer produced by Waters. Based on the height of the peaks from all ionic species of the peptide and complex the researchers calculated the binding affinity of tramiprosate to Aβ peptides and obtained the following results: 28 ± 3% for Aβ(1–42), 33 ± 1% for Aβ(1–40), 33 ± 3% for Aβ(1–28), 16 ± 2% for Aβ(1–28) all (−) and 0 for Aβ(1–28) all (+) [25].

Gervais and her collaborators demonstrated that tramiprosate forms non-covalent complexes with Aβ(1–40), Aβ(1–42) and Aβ(1–28) by infusing directly an aqueous solution containing 20 μM Aβ peptide alone or with 200 μM tramiprosate adjusted with 0.1% NaOH to the pH 7.4 ± 0.2 into the Micromass Q-TOF Micro mass spectrometer. Similarly, the researchers showed that taurine and N-acetylated homotaurine bind to Aβ(1–40) [26].

Kocis and his collaborators employed the mass spectrometer Synapt G2-S Q-TOF and performed ion mobility separation coupled to mass spectrometry for the characterization of Aβ(1–42) monomers and oligomers in the absence or presence of tramiprosate. Stock solutions of 5 mg/mL Aβ(1–42) and 1 mg/mL tramiprosate were prepared by separately dissolving 1 mg of these two substances in LC/MS grade water. Final solutions were prepared, containing 22 pmol/μL Aβ(1–42) in water alone or mixed with 10-, 100- or 1000-fold molar excess of tramiprosate. The samples were infused in the mass spectrometer after 0, 4 or 24 h incubation at room temperature. The analysis of the mass spectra recorded for the samples of Aβ(1–42) incubated for 24 h in the absence or in the presence of tramiprosate led to the conclusion that in the absence of tramiprosate, Aβ(1–42) solution contains monomers, dimers, trimers, tetramers, pentamers, hexamers and decamers. By comparison in the presence of a 1000-fold molar excess of tramiprosate, only monomers of Aβ(1–42) were observed. Additionally, the authors observed that the monomer of Aβ(1–42) was able to form noncovalent complexes with up to 6 molecules of tramiprosate and together with the fact that the drift times became shorter with the increasing number of bound tramiprosate molecules they concluded that the conformations of the Aβ(1–42) were fewer [27].

The treatment of primary rat neuronal cultures with a dose of 5 μM Aβ(1–42) has induced, 58.1 ± 3.4% dead neuronal cells after 72 h, while the treatment with a mixture of 100 μM tramiprosate and 5 μM Aβ(1–42) showed through a decrease in the number of dead neuronal cells (35.6 ± 5.2%) the protective activity of tramiprosate [26].

Similarly, daily subcutaneous therapy with tramiprosate in AD TgCRND8 transgenic mice (30 or 100 mg/kg over 8–9 weeks) revealed a statistically significant decrease of neocortical plaques load by 30%. Combined with the data according to which a reduction between 20–30% in brain levels of soluble and insoluble Aβ(1–40) and Aβ(1–42) occurred and that a dose-dependent decrease of around 60% in Aβ plasma levels took place, the hypothesis that tramiprosate influences the main deposit of Aβ by changing its brain efflux or catabolism emerged [25,26].

### 2.3. Melatonin

Bazoti and collaborators, using two mass spectrometers equipped with electrospray sources, reported in 2005 the formation of a noncovalent complex between Aβ(1–40) and melatonin, the pineal hormone. The researchers used an equimolar mixture of both substances at different concentrations and found that the concentration of 100 μM produced the most abundant signals of the complex formed between melatonin and Aβ(1–40). The results indicated that only one molecule of melatonin bound to one molecule of Aβ(1–40). In this study specific proteolytic enzymes (trypsin, chymotrypsin, Glu-C) were used in order to identify the binding amino acid sequence. When the enzymatic digestion was performed prior to the formation of the non-covalent complex the mixture analyzed at the mass spectrometer revealed the presence of various peptide fragments bound separately to one molecule of melatonin. When the non-covalent complexes were digested with proteolytic enzymes the mass spectra of the mixtures obtained indicated also that multiple proteolytic peptides bound separately to one molecule of melatonin. Incomplete digestion with trypsin prior or after the formation of the non-covalent complex led to the observation of two peptide fragments and one molecule of melatonin [28].

Investigations performed using murine N2a neuroblastoma demonstrated that melatonin (10 μM) had cytoprotective effect and prevented cell death when it was co-incubated with Aβ(25–35) (50 μM) [29]. 

An earlier report of experiments carried out in vivo on chronic melatonin treatment (0.5 mg/mL) started at 4 months of life and continued up to different time points (8, 9.5, 11 and 15.5 months) on Tg2576 mice was correlated with limited inhibition of the expected time-dependent elevation of Aβ, reduction of protein nitration and an improvement in survival [30]. Additionally, Quinn et al. [31] displayed that melatonin did not disturb the expression of APP holoprotein in the same Tg2576 mice model. Recently, Gong et al. [32] have revealed that melatonin treatment significantly attenuates cognitive deficits in Aβ(1–42)-induced mouse model. Doses of 10, 5 or 2.5 mg/kg melatonin were administered intraperitoneally for 14 days after 48 h from the icv injection of 410 pmol Aβ(1–42), dissolved in 5 μL PBS and inoculated in 2 min. Moreover, melatonin has reduced mitochondrial impairments and alleviated the expression of the phosphorylated-tau and several major proteins involved in apoptosis. These important results testify that melatonin has the capacity to control APP metabolism and inhibit Aβ pathology, but if it is administered after the development of Aβ deposits, it fails to have anti-amyloid or antioxidant properties. Furthermore, the in vivo results encouraged the use of melatonin and its analogs in the prevention of AD in high-risk populations [33].

### 2.4. Oleuropein

The complex formed by Aβ(1–40) and oleuropein, a polyphenol found in the fruits and leaves of *Olea europaea L.* was investigated. The peptide Aβ(1–40) was incubated with oleuropein at a 1:1 molar ratio by mixing identical volumes of 100 μM Aβ(1–40) freshly prepared in deionized water and of oleuropein solubilized in 1 mM ammonium acetate containing 0.5% acetic acid. The molecules were allowed to interact for different time intervals during 25 days prior to the extraction and direct infusion of a small sample aliquot into the SCIEX API III triple quadrupole mass spectrometer. The researchers identified in the mass spectra the signals corresponding to the molecular ions carrying 4 to 6 positive charges of the complex between one molecule of peptide and one molecule of oleuropein. They also determined experimentally the optimal pH value, electrospray source parameters and percentage of organic modifier establishing that the stoichiometry of the complex was neither influenced during the optimization of the solution concentrations of analytes, solvents or electrospray source parameters nor by addition of a ten-fold molar excess of oleuropein in comparison with amyloid beta (1–40) peptide [34].

**Figure 3:**
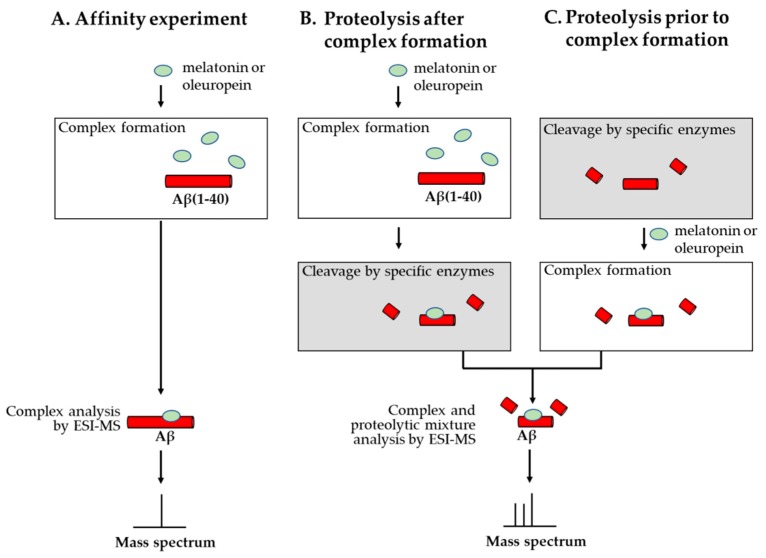
Schematic representation of the affinity mass spectrometric experiments carried out for the identification of the binding region from Aβ interacting with the test substances melatonin and oleuropein. (**A**) In the affinity experiment, incubation of Aβ(1–40) with melatonin or oleuropein in a suitable buffer for ESI-MS analysis indicates whether a noncovalent complex is formed. (**B**) Complex formation followed by cleavage with specific proteolytic enzymes and analysis of the complex together with the proteolytic mixture by ESI-MS allows the identification of the binding region from Aβ. (**C**) Proteolysis prior to complex formation and analysis of the mixture by ESI-MS allows the identification of the peptides that are incomplete and do not maintain affinity to the tested substance.

In a second study by the same team, proteolytic digestion using specific enzymes (trypsin and Glu-C) was performed. The resulting mixtures of peptides were analyzed using a Bruker Daltonics BioAPEX-94e superconducting 9.4 T FT-ICR mass spectrometer equipped with an electrospray ionization source and multiple proteolytic fragments bound separately to one molecule of oleuropein were observed in the mass spectra (Figure 3) [35].

To confirm the protective effect of oleuropein against Aβ(1–42)-induced toxicity, the viability of SH-SY5Y cells pre-incubated with different doses of oleuropein (10, 50, 250 and 1000 μM) for 24 h prior to the addition of a 25 μM solution containing fibrils of Aβ(1–42) was evaluated. The results indicated that in the absence of oleuropein, 25 μM Aβ(1–42), determines a cell viability of only 40%. After the pre-incubation with increasing concentrations of oleuropein the number of viable cells increased at 48% at 10 μM oleuropein and 68% at 1000 μM oleuropein [36]. 

Current data dealing with eight weeks of consumption of oleuropein aglycone in dietary therapy (50 mg/kg diet daily intake) for young-middle aged TgCRND8 and wild type mice offer convincing evidence that it improves the behavior of animals in step-down inhibitory avoidance and object recognition tests with regard to typically fed littermates. Enhanced behavior is sustained by a substantial reduction in Aβ(1–40) and Aβ(1–42) levels, in area and consistency of amyloid plaques and by the occurrence of soft deposits in the older mice [37]. 

The level of oleuropein aglycone to interplay with amyloid-β proteotoxicity in vivo by using the transgenic mice (TgCRND8) was evaluated by Pantano et al. [38]. Adding to the diet 12.5 mg/kg or 0.5 mg/kg of oleuropein aglycone an improvement in cognitive outcomes in two memory tests was recorded. Moreover, a reduction in the number and size of Aβ(1–42) plaques present in the brain cortex of the treated animals was found. In light of the above findings we can conclude that oleuropein aglycone compensates for Aβ aggregation and modulates the neurotoxicity attributed to the last of them and disturbs diverse pathways such as the processing of amyloid precursor proteins, the amyloid-β and tau protein aggregates, autophagy damage and neuroinflammation [39].

### 2.5. Trehalose

Wei Qi and collaborators reported in 2009 adducts formed by trehalose and Aβ(1–40) using concentrations of 100 μM peptide and disaccharide concentrations of 0.1 mM, 10 mM, 50 mM, 100 mM and 250 mM. At the concentration of 250 mM trehalose the number of disaccharide molecules which form adducts with one molecule of Aβ(1–40) is 9 [40].

Liu and the collaborators investigated the effects of different concentrations of trehalose on the toxicity exerted by Aβ(1–40) and Aβ(1–42) towards human neuroblastoma cells (SH-SY5Y). Fibrils of Aβ(1–40) and Aβ(1–42) were obtained by incubating these peptides over a four days and 12 h interval, respectively. Medium solutions containing fibrils of Aβ(1–40) and Aβ(1–42) at final concentrations of 800 nM and 400 nM respectively were prepared and added to the cells. At the addition of Aβ(1–40) the resulting cellular populations contained 64% viable cells in comparison with control cells and in the case of Aβ(1–42) only 62% viable cells. The addition of 0.1 mM, 1 mM, 10 mM or 50 mM trehalose at the beginning of the fibril formation step in the case of Aβ(1–40), the cellular viability increased in a dose-dependent manner up to 100 % at the highest trehalose concentration, however, showed no effect towards the toxicity exerted by Aβ(1–42) when added to the Aβ(1–42) solution at the beginning of fibril formation step. [41]

Trehalose treatment (2% solution) for AD Tg2576 transgenic mice (a daily oral dose of 0.1 mL/10g/b.w. daily for 31 days) remarkably increased performance in a behavioral test, corresponding with improved learning and memory. Improvement of cognitive outcomes was not correlated with a substantial modulation of Aβ peptide precursors [42]. Compelling findings have also been reported by Du et al., [43] in the treatment of AD APP/PS1 transgenic mice with trehalose (2 g/L of trehalose delivered into the right lateral ventricles). Cognitive impairment and learning deficits were ameliorated, while Aβ aggregates at the hippocampus and cerebral cortex were diminished. These reports support the theory by which trehalose can access a series of neural protective mechanisms to enhance the cognitive functions in AD that are independent of most trehalose-mediated pathways, including reduction of Aβ and autophagy activation. Moreover, trehalose appears to have a protective role in denaturing and conformational protein changes in some neurological diseases such as Alzheimer’s and Parkinson’s disease [44].

### 2.6. β-cyclodextrin

Camilleri and his collaborators investigated in 1994 the interaction between Aβ(1–40) and β-cyclodextrin using a mass spectrometer equipped with an electrospray source and a single quadrupole mass analyzer. Although Aβ(1–40) peptide and β-cyclodextrin were solubilized in a solvent containing water, methanol and acetic acid which are denaturing experimental conditions, a strong signal corresponding to the quadruply charged ion of the complex between one molecule of β-cyclodextrin and one molecule of Aβ(1–40) was observed in the mass spectrum [45].

β-Cyclodextrin has proven to reduce the neurotoxicity of Aβ(1–40) towards PC12 cells (rat phaeochromocytoma) in a dose dependent manner. At the maximum dose (23 µM) of Aβ(1–40), the cytotoxic effect was of approximately 55%, while the incubation of the PC12 cells with both Aβ(1–40) and β-cyclodextrin (75 µM) led to a reduction in cell viability of only 30% at 24 h [45].

Investigation of the potential protection role of 2-hydroxypropyl-β-cyclodextrin (HP-β-CD), a β-cyclodextrin derivative possessing higher solubility in aqueous solutions, was based on viability assessment into SH-SY5Y cells (human neuroblastoma). In the control group, in which pure HP-β-CD (125 μm) was added, no signs of cytotoxicity were registered and cell viability at 48 h in cell culture was of 98.8%. The pure Aβ was highly toxic to cells, whose viability was dramatically decreased to 48% at 48 h, causing morphological changes (cell body shrinkage and aggregation). When incubating HP-β-CD with Aβ(1–42), HP-β-CD was able to protect cells from the Aβ-induced cell toxicity. The viability at different ratios (1:1, 1:2, and 1:5) of added Aβ-HP-β-CD mixture varied between 74%, 93% and 82% at 24 h greater than that of Aβ alone (60%). Further increase of incubation time to 48 h led to continuous, but overall minor decrease in cell viability to 61%, 91%, and 75% at 1:1, 1:2, and 1:5 ratios, respectively, suggesting that HP-β-CD can retain its long-term neuroprotection against Aβ-induced toxicity in SH-SY5Y cells, especially at 1:2 molar ratio which demonstrated the most potent protective effect [46].

The therapeutic properties of HP-β-CD were evaluated on an AD Tg19959 mice model by Yao et al., [47]. Subcutaneous administration of 4.000 mg/kg HP-β-CD compound twice a week over a 4-month period beginning at 7 days of age resulted in an improvement over memory impairments and spatial learning, reduction of the Aβ aggregates production as well as tau cerebral cortex and hippocampus immunopositive dystrophic neuritis. Moreover, as a consequence of reducing the cleavage of APP protein and upregulation for the expression of some genes involved in the transport of cholesterol, a decrease in amyloid aggregates in the brains of mice was observed. HP-β-CD has a direct action on intensifying cholesterol transport and Aβ clearance through ABCA1 modulation. In an AD model with Npc1-deficient mice, HP-β-CD therapy increased the lifespan and ameliorated behavioral deficits and pathological abnormalities accompanying the experimental model [48].

HP-β-CD short-term therapy (two weeks) on autophagy in vivo, on wild-type and adult/old TgCRND8 mice in varying concentrations over the three routes of administration (ip delivery: 4000 mg/kg/day 20% HP-β-CD solution; icv: 40 mg/kg/day, 2 μL of 40% solution of HP-β-CD, or intrahippocampal injection: single dose of 0.06 or 0.12 mg, 0.3 μL of 20 or 40% HP-β-CD solution) has been evaluated. The outcomes revealed that icv delivery of HP-β-CD significantly reduced the dimensions of enlarged autolysosomes and dropped the GM2 ganglioside and Aβ-immunoreactivity without changing APP proteins processing, extracellular Aβ or β-amyloid burden. The main mechanisms by which HP-β-CD acts on autophagy are represented by stimulation of lysosomal proteolytic activity by growing cathepsin D action and by preventing autophagosome-lysosome synthesis [49].

### 2.7. GRKKRRQRRR-GGGG-DVEFRH Peptide

Cimini and her collaborators employed a MALDI-TOF/TOF 4800 mass spectrometer produced by Applied Biosystem for the MALDI-imaging mass spectrometric investigation of 15 μm thick slices of brain obtained from mice sacrificed after 6 and 24 h from the intraperitoneal administration of the peptide designated Aβ(1–6)A2VTAT(D). The amino acid sequence of this peptide is GRKKRRQRRR-GGGG-DVEFRH and the amino acids used for its synthesis were all d-FMOC amino acids. The authors observed abundant peptide signals in the brain slices obtained 6 h after treatment as compared with less abundant peptide signals in the brain slices collected 24 h after administration and concluded that the peptide crossed the blood brain barrier [50].

Treatment of SH-SY5Y cells with Aβ(1–6)(D) or Aβ(1–6)A2V(D) showed that neither is toxic for living cells even at high concentrations (20 μM) and that both peptides can reduce the toxicity induced by Aβ(1–42). However, Aβ(1–6)A2V(D) showed a stronger effect in counteracting the reduction of cell viability caused by Aβ(1–42) (35%), suggesting that the A-to-V substitution actually amplifies the protective effects of the six-mer peptide. 

Aβ(1–6)A2VTAT(D) is not toxic when added to culture medium at concentrations ranging between 1 and 5 μM, while it reduces cell viability at higher concentrations. Aβ(1–6)A2VTAT(D) showed a dose-dependent effect in reducing Aβ(1–42) toxicity [51]. Investigation of Aβ(1–6)A2VTAT(D) cytotoxic impact on primary hippocampal neurons did not induce neuronal death at any of the concentrations tested (2–20 µM) after 24 h incubation, as assessed through MTT and LDH assays. Also, Aβ(1–6)A2VTAT(D) peptide reverted soluble Aβ(1–42) toxicity in vitro [50].

An in vivo report performed on AD APPswe/PS1dE9 mice revealed that short-term therapy (2.5 months) with 10 mg/kg Aβ(1–6)A2VTAT (D) ip administration weekly, results in a decrease of Aβ production and accumulation, with inhibition of amyloid deposition in all cerebral areas. Moreover, there was an augmentation in the soluble fraction of Aβ(1–42)associated with a reduction of the insoluble compartment, indicating that the therapy may lead to a transfer of Aβ(1–42) from the insoluble to soluble fraction of cerebral tissue, which may consider a disruption of amyloid deposits. Instead, a prolonged therapy (5 months) leads to an unexpected augmentation of amyloid burden even if it avoids cognitive impairment [51]. The unwanted results of the long-term treatment with Aβ(1–6)A2VTAT (D) might be determined by the TAT intrinsic ability to induce an increase in Aβ production, phosphorylation of tau and then neuronal death during AD development [52].

Evaluating the synaptotoxic effect of some oligomers formed by the aggregation of various Aβ peptides, revealed that a single ip injection (20 mg/kg) of Aβ(1–6)A2VTAT (D) administered to the TgCRND8 mice ameliorated the postsynaptic alterations of GluN2A, GluA1, GluA2 values and the scaffold PSD-95 level was considerably augmented. These findings suggest that Aβ(1–6)A2VTAT (D) peptide in some measure contrasts AD synaptopathy [50].

### 2.8. Ac-VVIA and VVIA-NH_2_ Peptides

**Figure 4:**
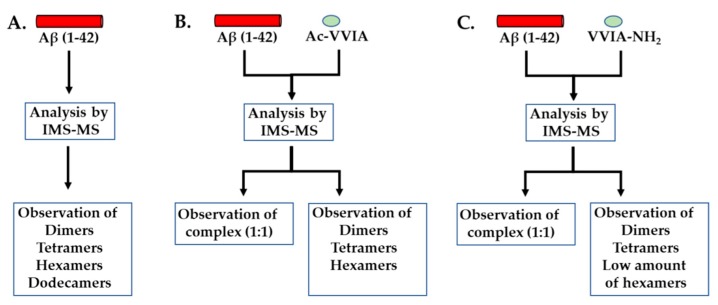
Schematic representation of the ion mobility spectrometry-mass spectrometry experiments performed for the investigation of Aβ(1–42) oligomerization in the absence (**A**) or presence of the tetrapeptides Ac-VVIA (**B**) and VVIA-NH2 (**C**).

Two four amino acid long peptides corresponding to the carboxy-terminal end of the peptide Aβ(1–42) were synthesized for testing the binding to the Aβ(1–42) peptide and the disaggregating effects on preformed oligomers; the first one acetylated at the amino-terminal end Ac-VVIA and the second one possessing an amidated carboxy-terminal end VVIA-NH_2_ [53]. Using an ion mobility spectrometer–mass spectrometer the researchers observed that a 10 μM solution of Aβ(1–42) prepared in a 10 mM ammonium acetate buffer solution having the pH 7.4 yielded dimers, tetramers, hexamers and dodecamers. The addition of the peptide Ac-VVIA during the formation of the oligomers caused the disappearance of the Aβ(1–42) dodecamers. The incubation of the peptide Aβ(1–42) with the peptide VVIA-NH_2_ resulted in a diminished concentration of the hexamer and the absence of the dodecamer. Moreover the mass spectra indicated the presence of complexes between each of the two tetrapeptides and the Aβ(1–42) peptide (Figure 4).

Evaluation of cytotoxicity of VVIA-NH_2_ in PC-12 cells concluded that VVIA-NH_2_ did not induce toxic effects, by contrary causing a significant increase of 10–35% in cell viability compared to control cells. Investigation of VVIA-NH_2_ capacity to inhibit Aβ42-induced neurotoxicity in single-dose experiments, the PC-12 cells were incubated with Aβ42 for 24 h in the absence or presence of 10-fold excess of VVIA-NH_2_, proving its capacity to significantly attenuate the Aβ42-induced toxicity. The Ac-VVIA showed no inhibitory activity [54].

### 2.9. RYYAAFFARR Peptide

Liu and his collaborators investigated the binding to Aβ(1–40) of the peptide Ac-RRYYAAFFARR-NH_2_ using surface plasmon resonance assay and carried out MALDI-TOF mass spectrometric analysis of the solution containing Aβ(1–40) which was prepared for immobilization on the sensor chip. The mass spectrum indicated that the amyloid beta peptide did not form aggregates. The authors verified also by MALDI-TOF mass spectrometry whether the decapeptide yields oligomers when incubated at a concentration of 20 μM in 10 mM PBS (pH 7.4) during 24 h and concluded that the peptide does not form aggregates [55].

RYYAAFFARR decapeptide inhibitor (RR) has proven to be an efficient protector of PC-12 cells (highly differentiated rat pheochromocytoma) against Aβ(1−40)-induced toxicity, but to different extents. Cell viability after Aβ(1−40) (20 μM) treatment was approximately 65% that of the control, whereas incubation of Aβ1−40 with RR enhanced cell viability. Furthermore, RR in a molar ratio of 1:1 (Aβ(1−40)/RR) was sufficient to significantly prevent Aβ(1−40)-induced toxicity and Aβ(1−40) aggregation. The viability of PC-12 cells increased to about 95% when Aβ(1−40) was incubated with RR in a molar ratio of 1:4 (Aβ(1−40)/RR), which was almost equal to the control. Because Aβ neurotoxicity is related to the formation of the β-sheet structure, the MTT assay results suggest that RR is an effective inhibitor of Aβ(1−40) [55].

The bifunctional Aβ aggregation inhibitor GGHRYYAAFFARR (GR), in which RYYAAFFARR (RR) acted as an aggregation inhibitor and GGH had chelator function for Cu(II) ions, was investigated in regards to cytotoxicity over neuronal PC12 cells (highly differentiated rat pheochromocytoma) by MTT assay. The addition of Aβ fibrils-Cu complex and different transformation solutions with the same concentration of Aβ to the cells, showed at 48 h an enhanced toxicity for fAβ-Cu (cell viabilities of 38%), due presumably to the toxicity of β-sheet structure content and ROS generated by Cu in the presence of ascorbate [56]. The addition of GGH or RR has increased the cell survival rate by up to 46% and 48%, respectively, being to only suppress the toxicity of just one aspect: GGH has merely reduced the ROS toxicity induced by Cu, whereas RR has only decreased the β-sheet structure within aggregates. Thus, the cell viability has not been largely improved. The cell viability in the group treated with GR has greatly increased up to 70%, through the combined effects of the chelator GGH (46%) and single functional peptide RR (48%). This is a strong evidence of the excellent potential of GR toward Cu ions chelation and Aβ degradation [56].

Another study investigated the capacity of GR on inhibiting the cytotoxicity of Aβ-Cu(II) complex. The cell viability of the Aβ group was 62% and the addition of Cu(II) ions greatly reduced cell viability to 35%. When GGH was added, the cell viability could be significantly improved to 49% because of its ability to chelate Cu(II) ions and, in turn, inhibit Aβ aggregation. When GR, which has the ability to chelate Cu(II) ions and has anti-amyloid effect, was added, the cell viability improved to 88%, significantly better than that of the RR group (68%). This improvement illustrated the properties of the bifunctional inhibitor in reducing the cytotoxicity of Aβ-Cu(II) complex and its advantage over the single functional inhibitors GGH and RR [57].

### 2.10. RGTFEGKF Peptide

Barucker and his collaborators analyzed the binding region from Aβ(1–42) involved in the interaction with the peptide RGTFEGKF (named also AIP) using a UltrafleXtreme MALDI-TOF/TOF produced by Bruker Daltonics. The authors verified whether the peptides RGTFEGKF containing all l or all d amino acids are cleaved by trypsin and found that after 3 h incubation with trypsin there were no fragments in the mass spectra. They also proved that both octapeptides decreased the cleavage by trypsin of the peptide Aβ(1–42) after the lysine-28 by comparing the ratio between the area under curve obtained for each of the tryptic fragments Aβ(1–16) and Aβ(17–28) and for Aβ(1–42) [58].

The investigation of AIP effects on neurotoxicity was assessed in SH-SY5Y cells. In the absence of AIP, freshly dissolved Aβ(1–42) peptides (pre-incubated for 4 or 8 h) reduced the number of living cells by approximately 40%; under similar assay conditions, the presence of AIP was able to neutralize the Aβ(1–42)-induced neurotoxic effects. In the case of primary hippocampal neurons that were pre-incubated with Aβ(1–42), AIP almost completely attenuated the Aβ(1–42)-mediated loss of living cells at 4 h’ time interval [7,58].

## 3. Conclusions

**Figure 5:**
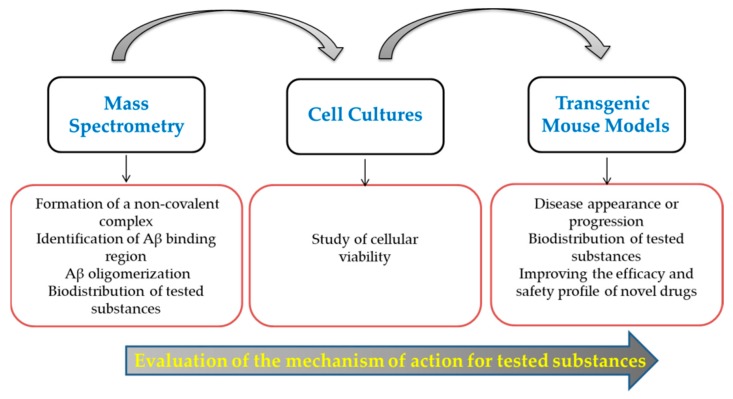
Workflow for the evaluation of the mechanism of action for potential inhibitors of Aβ oligomerization.

The verification by mass spectrometry of non-covalent complex formation between amyloid-beta peptides and the substances tested in vitro and in vivo for lowering the toxic effect of the amyloid-beta peptides is an important step for clarifying the mechanism of action for these substances (Figure 5).

Further studies should also use the recently developed method namely Intact Transition Epitope Mapping (ITEM) which allows a rapid analysis of an epitope recognized by an antibody and which could be applied to non-covalent complexes between low molecular weight molecules [59]. For additional information on the importance of each amino acid residue from the Aβ region involved in complex formation with the substance analyzed peptides containing alanine single site mutations within Aβ binding sequence could be produced by solid phase peptide synthesis and the complex formation could be investigated for each mutant peptide by mass spectrometry [60].

## Abbreviations

AD, Alzheimer’s Disease; Aβ, Amyloid-beta; APP, Amyloid Precursor Protein; FAD, Familial Alzheimer’s Disease; ABCA1 modulation, ATP binding cassette transporter A1; AD model with Npc1-deficient mice, a novel mouse model of “Juvenile Alzheimer’s Disease” carrying a D1005G-Npc1 mutation; GluA1 and GluA2 subunits of α-amino-3-hydroxy-5-methyl-4-isoxazolepropionic acid (AMPA) receptors; GluN2A, the main subunit of N-methyl-d-aspartate receptors; PSD-95, a membrane-associated guanylate kinase, is the major scaffolding protein in the excitatory postsynaptic density (PSD) and a potent regulator of synaptic strength; ESI, Electrospray ionization; MALDI, Matrix Assisted Laser Desorption and Ionization; iPSCs, Induced pluripotent stem cells; PS1, Presenilin 1; PS2, Presenilin 2; SH-SY5Y, Human neuroblastoma cell line; GAP-43, Growth-Associated Protein; NeuN, Neuronal Nuclei; SYN, Synaptophysin; SV2, Synaptic Vesicle Protein II; NSE, Neuron Specific Enolase; MAP, Microtubule Associated protein; GFAP, Glial Fibrillary Acidic Protein; SK-N-SH, Original Human Neuroblastoma Cell Line from which SH-SY5Y was derived; TH, Tyrosine Hydroxylase; GSK3 beta, Glycogen synthase kinase 3 beta; HN, Humanin peptide; Acm, Acetamidomethyl; NHS-Activated Sepharose, N-hydroxysuccinimide—Activated Sepharose; MALDI-TOF, Matrix Assisted Laser Desorption and Ionization—Time Of Flight; nano-ESI-FTICR, Nano-Electrospray Ionization—Fourier Transform Ion Cyclotron Resonance; PC12, Rat phaeochromocytoma; Bcl-2, B-cell lumphoma 2, antiapoptotic protein; Bax, Bcl-2-like protein 4, proapoptotic protein, Bax gene; icv, intracerebroventricular; ip, intraperitoneal; APPswe/PS1dE9 mice, double transgenic mouse model carrying APP Swedish mutation and deletion of presenilin 1 in exon 9; APPswe, tauP310L, PS1M146V mice, triple transgenic mouse model carrying APP Swedish mutation, Tau mutation P310L and presenilin 1 mutation M146V; 3xTg-AD mice, triple transgenic mouse model of Alzheimer’s disease; Q-TOF, Quadrupole—Time Of Flight; LC/MS, Liquid Cromatography/Mass Spectrometry; Glu-C, Endoprotease V8 from Staphylococcus aureus; N2a, mouse neuroblastoma cell line; Tg2576 mice, transgenic mice overexpressing APP695 carrying Swedish mutation K670N and M671L; TgCRND8 mice, transgenic mouse model of Alzheimer’s Disease overexpressing APP695 and carrying Swedish mutation K670N and M671L and Indiana mutation V717F; HP-β-CD, 1-hydroxypropyl-β-cyclodextrin; Tg19959 mice, transgenic mouse model of Alzheimer’s disease, which overexpress human APP with the Swedish and Indiana familial AD mutations; Fmoc, 9-Fluorenyl- methyloxycarbonyl; MTT, 3-(4,5-dimethylthiazol-2-yl)-2,5-diphenyltetrazolium bromide; fAβ-Cu, Amyloid- beta fibrils Cu complex; ROS, Reactive Oxygen Species.
